# Obesity and Asthma: A Missing Link

**DOI:** 10.3390/ijms18071490

**Published:** 2017-07-11

**Authors:** Mª Amelia Gomez-Llorente, Raquel Romero, Natalia Chueca, Ana Martinez-Cañavate, Carolina Gomez-Llorente

**Affiliations:** 1 Pediatric Unit, Hospital Materno-infantil, Ciudad sanitaria Virgen de las Nieves, 18014 Granada, Spain; mariaa.gomez.sspa@juntadeandalucia.es; 2 Investigación Biosanitaria ibs, 18012 Granada, Spain; naisses@yahoo.es; 3 Pediatry Unit, San Cecilio University Hospital, 18012 Granada, Spain; rakelina20@hotmail.com; 4 Departmennt of Microbiology, Complejo Hospitalario de Granada, 18012 Granada, Spain; 5 Pediatric Allergology, Hospital Materno-infantil, Ciudad sanitaria Virgen de las Nieves, 18014 Granada, Spain; anamartinezcanavate@gmail.com; 6 Department of Biochemistry and Molecular Biology II, School of Pharmacy, Institute of Nutrition and Food Technology “José Mataix”, Biomedical Research Center, University of Granada, 18100 Armilla, Spain; 7 CIBEROBN (Physiopathology of Obesity and Nutrition CB12/03/30038), Instituto de Salud Carlos III (ISCIII), 28029 Madrid, Spain

**Keywords:** obesity, asthma, gastrointestinal microbiome, adipokines

## Abstract

Obesity and asthma are two chronic conditions that affect millions of people. Genetic and lifestyle factors such as diet, physical activity, and early exposure to micro-organisms are important factors that may contribute to the escalating prevalence of both conditions. The prevalence of asthma is higher in obese individuals. Recently, two major phenotypes of asthma with obesity have been described: one phenotype of early-onset asthma that is aggravated by obesity, and a second phenotype of later-onset asthma that predominantly affects women. Systemic inflammation and mechanical effect, both due to the expansion of the adipose tissue, have been proposed as the main reasons for the association between obesity and asthma. However, the mechanisms involved are not yet fully understood. Moreover, it has also been suggested that insulin resistance syndrome can have a role in the association between these conditions. The intestinal microbiota is an important factor in the development of the immune system, and can be considered a link between obesity and asthma. In the obese state, higher lipopolysaccharide (LPS) serum levels as a consequence of a microbiota dysbiosis have been found. In addition, changes in microbiota composition result in a modification of carbohydrate fermentation capacity, therefore modifying short chain fatty acid (SCFA) levels. The main objective of this review is to summarize the principal findings that link obesity and asthma.

## 1. Introduction

**Figure 1:**
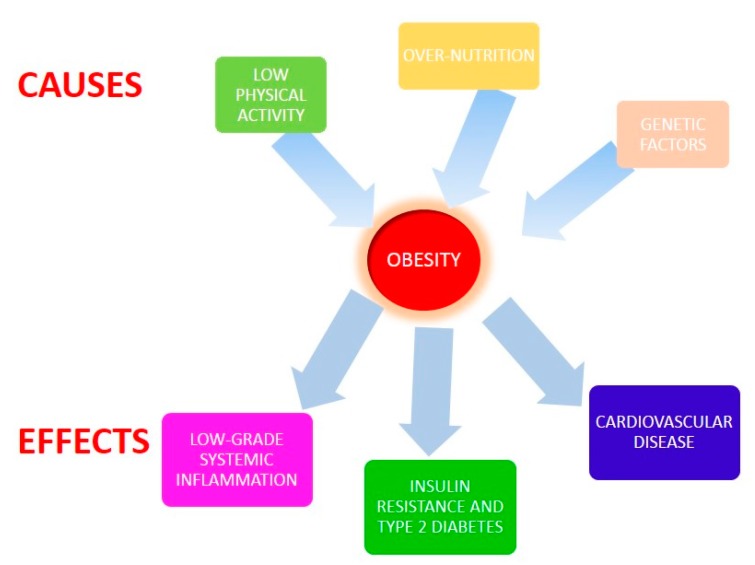
Causes and effects of obesity.

Obesity is considered one the most important public health problems of the 21st century. According to the World Health Organization, in 2014, more than 1.9 billion adults were overweight, and 41 million children under the age of 5 were overweight or obese [1]. Genetic and lifestyle factors, mainly over-nutrition (an excess of carbohydrate and fat ingestion and a low ingestion of vegetables, fruits, and whole grains) and low physical activity are important factors that contribute to the escalation of this condition [2]. Obesity is designated by an increased body mass index (BMI) and by systemic low-grade inflammation. In addition, obesity is associated with the development of type 2 diabetes and with cardiovascular and non-alcoholic fatty liver disease risk [3] (Figure 1).

Asthma is a chronic inflammation disorder, but of the airways linked with airway hyper-responsiveness (AHR), and is characterized by recurrent episodes of wheezing, breathlessness, chest tightness, and coughing. These episodes are usually associated with widespread, but variable, airflow obstruction within the lung that can be reversible either spontaneously or with treatment [4]. The Global Asthma Report estimated that 334 million people have asthma based on the analyses of the Global Burden of Disease Study (GBD) undertaken in 2008–2010. Asthma usually begins in childhood, and is often associated with other allergic disorders (in other words, atopic dermatitis and seasonal rhinitis). Inflammation includes T helper 2 CD4 T (Th2) cells and associated cytokines plus eosinophilic infiltration. Eosinophils are the effector cells in allergic inflammation and contribute to the production of interleukin (IL) 4 and IL-13, which are necessary for increased muscle reactivity [5].

In 1999, Camargo et al. [6] described for the first time the relationship between obesity and asthma. Since then, a huge number of studies have demonstrated the risk of asthma and associated symptoms in obese individuals, in both children and adult populations [7]. The asthma phenotype in obese individuals is characterized by more severe symptoms, destabilization or lack of asthma control, worse quality of life, lack of eosinophilic inflammation, different responses to controller medication, and the development of steroid resistance [8]. Currently, two different obese-asthma phenotypes have been described: an allergic early-onset disease and a non-allergic late-onset disease [9]. The mechanisms linking obesity and asthma are poorly understood. Genetics, lifestyle (diet, physical activity), and early exposure to micro-organisms are important factors that may contribute to the escalating prevalence of both conditions. Obesity-related low-grade inflammation originates from the adipose tissue, which is capable of secreting a variety of factors, such as ILs and adipokines, which are involved in glucose and lipid metabolism, angiogenesis, reproduction, immunity, and in body weight control [10,11]. Among these factors, leptin and adiponectin have been proposed as key molecules linking obesity and asthmatic conditions. The adipose tissue is comprised of different kinds of cells, among which the immune cells have an important role in the obesity-related low-grade inflammatory state [11]. On the other hand, the hygiene hypothesis states that exposure to specific micro-organisms and parasites in babyhood has known benefits on immune system development and supply protection for autoimmune diseases. One explanation for this effect is that removing microbes from individuals’ living environments may alter their indigenous intestinal microbiota, the micro-organisms that inhabit the gastrointestinal tract [12]. A dysbiosis of the intestinal microbiota has been associated with a number of human diseases, such as autoimmune diseases, obesity, and asthma. In the obese state, higher lipopolysaccharide (LPS) serum levels because of microbiota dysbiosis have been found. In addition, changes in the microbiota composition can affect the body’s capacity to produce short chain fatty acids, as well as to affect the metabolism of bile acids.

Overall, the aim of the present work is to carry out a revision of the scientific evidence on the obesity-asthma relationship, and to gain a better understanding of the underlying mechanisms and the putative role of visceral adipose tissue and gut microbiome.

## 2. Results

### 2.1. Asthma-Associated Obestiy Phenotypes

Numerous studies support an association between a higher BMI and adiposity with asthma in both adult and pediatric populations [13,14]. Some of them have described a more positive association in adult women compared with men; however, epidemiologic studies of children have provided mixed results [15]. It has been shown that asthma prevalence in children increases with BMI, but only in the obese and morbidly obese range. Moreover, in this population, asthma is directly associated with elevated serum triglyceride levels and insulin resistance [16]. On the other hand, it has been shown that in patients who are severely obese, bariatric surgery improved asthma severity and control, airway responsiveness, and lung volumes [17]. In the same way, based on Global Initiative for Asthma (GINA) criteria, for patients with uncontrolled asthma and with moderate obesity, weight loss was related with enhancements in asthma control. This result was accompanied by increased forced vital capacity (FVC), but not by alterations in airway inflammation or bronchial reactivity markers [18]. Moreover, this study also suggested that poor asthma control is, at least in part, related to obesity-associated factors. It is important to highlight that the group of patients was formed primarily of women with moderate obesity and early-onset severe asthma [18].

Although there is a clear relationship between obesity and asthma, there are a number of subjects who are obese with a misdiagnosed asthma. The main difference found between misdiagnosed asthma subjects and control subjects, both of them with obesity, was a higher perception of dyspnea during bronchial challenge and exercise [18]. Moreover, in subjects who are obese, the perception of dyspnea during exercise was independent of peak respiratory frequencies, peak ventilatory equivalent of CO_2_, and serum levels of IL-6 and IL-1β [19].

The connection between obesity and asthma is so stunning that subjects who are obese with asthma are considered a new asthma phenotype. Two distinct subgroups of obese asthmatics have been described: an early-onset atopic asthma Th2-high, where allergic asthma is complicated by the presence of obesity, and a late-onset non-atopic asthma Th2-low, occurring preferably in women and where the development of asthma is a consequence of obesity [20]. In the early-onset phenotype, obese asthmatics have a history of atopy, increased airway obstruction, greater bronchial hyper-responsiveness, higher Immunoglobulin E (IgE) levels, and a greater likelihood of allergic sensitization and reactions compared with late-onset obese asthmatics. In contrast, late-onset obese asthmatics had less atopy, less bronchial hyper-responsiveness, less airway obstruction, and fewer exacerbations compared with early-onset obese asthmatics [20].

There is a clear association between obesity and asthma, and probably childhood obesity antedates the onset of asthma. However, more studies that clarify the characteristics of the two described phenotypes are needed.

### 2.2. Systemic Inflammation and Adipokines in Asthma-Associated Obesity

Obesity is a complex disease, characterized by a systemic inflammatory state caused, at least in part, by the different adipokines secreted by the adipose tissue. In line with this, in obese subjects there is a rise in the serum concentrations of the pro-inflammatory adipokine leptin, which is structurally related to IL-6 and is the main regulator of appetite, whereas adiponectin levels are decreased. [21]. Adiponectin is synthesized and secreted by the adipocyte, regulates glucose and fatty acid metabolism, and also has anti-inflammatory properties. In patients who are obese, adipose tissue hypertrophies and becomes infiltrated with proinflammatory macrophages. The adipocytes and activated macrophages produce increased proinflammatory adipokines and cytokines that together with the decreased adiponectin levels generate “metabolic inflammation” [22]. For instance, it has been shown that markers of metabolic inflammation, mainly in visceral adipose tissue, are significantly higher in obese patients with late-onset asthma compared with control subjects [22]. While there is clear evidence of increased systemic inflammation in adults who are asthmatic-obese, there is conflicting evidence regarding systemic inflammation in children who are asthmatic-obese [23].

Macrophages in the adipose tissue play an important role in the pathogenesis of metabolic inflammation. Increased macrophage infiltration of visceral adipose tissue has been described in patients who are asthmatic-obese when controlled for BMI [22]. Recently, it has been shown that children who are asthmatic-obese have sex-specific macrophage activation, assessed by measuring soluble Cluster of differentiation (CD) 163, which may contribute to worse asthma control and lung function [23]. In addition, it has been shown that monocyte/macrophage programming is altered in adult patients who are obese and asthmatic. Efferocytosis by airway macrophages, a phagocytic process by which dying or dead cells are removed, was 40% lower in patients who were asthmatic-obese than in patients who were nonobese asthmatics [24]. On the other hand, an increased efferocytic index was observed in a control group of obese subjects compared to nonobese control subjects, both of them nonasthmatic [24]. Accordingly, it has been shown that children who were obese, asthmatic, and, asthmatic-obese present significant lower circulating regulatory T cells (Tregs) compared to healthy controls. Tregs have been shown to encourage neutrophil apoptosis, improve efferocytosis, and produce specific cytokines [25].

Additionally, limited lung function and increased airway hyper-reactivity have been reported in obese patients, and have been suggested as possible mechanisms linking obesity and asthma [26]. In mice without asthma, it was found that AHR or eosinophilic inflammation in lung tissue was not augmented by obesity (diet induced) per se [26]. However, in atopic asthmatic children and adolescents, obesity has been associated with increased serum leptin and tumor necrosis factor alpha (TNF-α) levels that enhance eosinophil chemotaxis and adhesion [27]. An inhibition of the activation of dendritic and T cells after the induction of respiratory tolerance and allergic airway inflammation in mice fed a high fat (HFD) diet for 8 weeks has recently been described. Mice fed a HFD had a marked decrease in Th2 cytokine production after the induction of allergic airway inflammation. However, these effects on pulmonary immune function and respiratory tolerance did not decrease lung inflammation and IgE production. Taken together, these results suggest that HFD tends to impair the response of airway immune cells to allergens, but does not alter the development of respiratory tolerance [28].

Epidemiological studies show an inconsistent relationship of adipokines with asthma, which in some studies appears to be gender specific [29]. Different studies have described elevated leptin levels in asthmatic children and adults [30,31]. However, although leptin levels were associated with asthma, it was not possible to rule out the possibility that this association was secondary to the association of both with fatness measures [31]. Regardless, leptin has been shown to increase the expression of pro-inflammatory cytokines TNF-α and IL-6, both associated with a Th2 phenotype, in adipocytes, macrophages, and T lymphocytes [32]. It has consistently been shown that the mRNA levels of TNF-α and IL-6, which have been implicated in the pathogenesis of atopic dermatitis, are higher in atopic obese rats than in the nonobese ones. Leptin might also induce allergic inflammation by the activation of eosinophils via altered expression profiles of chemokines and cytokines [32]. An adipocyte-dependent regulation of the bronchial diameter, the disruption of which contributes to impaired lung function caused by abnormal body weight, has been described in mice. Indeed, leptin increases airway diameter via its cognate receptor in cholinergic neurons, in a mechanism independent of its regulation of appetite, melanocortin pathway, or sympathetic tone [21]. Additionally, a significant relationship between fat leptin expression and airway reactivity has been reported [22]. All of these observations point to an important role for leptin in asthma-associated obesity.

Adiponectin is another important adipokine secreted by the adipocytes, levels of which have been reported to be lower in obese patients and to have an anti-inflammatory role, as previously mentioned. It has been reported that human bronchial epithelial cells are also capable of expressing adiponectin; in these cells, adiponectin probably acts as an endogenous repair promoter, antioxidant, and anti-apoptotic agent against ozone [33]. In the asthma context, it appears that adiponectin does not protect against the development of inflammation, and may in fact exacerbate the disease via its anti-Th1 inflammatory effects, allowing for increased Th2 differentiation and a more severe allergic response [34]. Although an association between adiponectin and pulmonary function has been described, there is conflicting data regarding the relationship between adiponectin levels and the presence of asthma, indicating that serum levels may not accurately reflect levels of adiponectin in the lungs [33]. The visceral adipose tissue of asthmatic-obese patients had significantly lower adiponectin at the time of bariatric surgery, while 12 months after the surgery the levels tended to be higher in subcutaneous adipose tissue [22]. Another pro-inflammatory adipokine is resistin, a protein that belongs to the family of resistin-like molecules [35]. Resistin levels and the resistin:adiponectin ratio have been found to be higher in asthmatic subjects than in control subjects. Among subjects with asthma, resistin levels between obese and nonobese males and females did not differ [35]. Moreover, it has been shown that asthma is associated with higher resistin levels and resistin:adiponectin ratios, and that these biomarkers were again higher in asthmatics with more severe disease. Moreover, asthmatic patients had lowered lung function, a higher rate of atopy, and a higher BMI. In addition, the resistin:adiponectin ratio was higher in asthmatic-obese males. Therefore, it has been suggested that resistin may be contributing to the obese-asthma phenotype, a possibility reinforced by the fact that reduction in body fat mass also reduces the resistin:adiponectin ratio [35].

To summarize, low-grade systemic inflammation is one possible mechanism linking obesity and asthma, but is unlikely to fully explain the obesity-asthma association, although TNF-α, IL-6, C-reactive protein (CRP), circulating neurotransmitter neuropeptide Y (NPY), and adiponectin levels have been independently associated with asthma prevalence [36]. Therefore, it has been proposed that the obesity-asthma relationship is more likely to be mediated by other factors, which include type 2 diabetes among others [36]. Obesity is an important risk factor for insulin resistance syndrome, also known as metabolic syndrome, a complex disorder comprised by a cluster of factors that include hypertension, altered energy metabolism, and low-grade inflammation. Positive associations with lung function impairment have been described for components of the metabolic syndrome [37]. In postmenopausal women, the incidence of metabolic syndrome was similar for asthma and control groups, but insulin resistance was significantly higher in asthma patients [38]. Nevertheless, patients who were obese and underwent preoperative screening for bariatric surgery presented a higher proportion of blood monocytes and eosinophils, and a lower forced expired volume in 1 second (FEV_1_)/forced vital capacity (FVC) ratio, indicating airway obstruction as compared to patients who were obese without metabolic syndrome [37]. Accordingly, the authors suggested that the presence of metabolic syndrome may influence lung function impairment through the induction of systemic inflammation, particularly inflammation mediated by blood eosinophils [37]. Recently, it has been shown that Th1 polarization and monocyte activation among obese-asthmatic adolescents correlates with metabolic abnormalities. The association of monocyte activation with pulmonary function is mediated by BMI, whereas that of Th1 polarization is mediated by insulin resistance [39]. Hence, type 2 diabetes and the chronic inflammation related to obesity are associated with an increase in cellular expression and plasma concentrations of mediators involved in the pathogenesis of asthma. Furthermore, following gastric bypass surgery and weight loss, there was a significant reduction in these mediators, including in IL-4, the main Th2-cytokine related to asthma [40]. Likewise, the suppression of the clinical activity of asthma by a preparation of a monoclonal antibody against the alpha subunit of IL-4 has been described [41]. In this regard, the intravenous infusion of insulin leads to the suppression of IL-4 expression in mononuclear cells from obese patients with type 2 diabetes. Moreover, insulin also suppresses the expression of ADAM metallopeptidase domain 33 (ADAM-33), TNF superfamily member 14 (LIGHT), and lymphotoxin beta receptor (LTBR), and reduces the plasma concentrations of plasma concentration of nitric oxide metabolites (NOM), LIGHT, transforming growth factor beta 1 (TGF-β1), C–C motif chemokine ligand 2 (MCP-1), and matrix metallopeptidase 9 (MMP-9), all associated with asthma [42]. These data correspond to the anti-inflammatory effects of insulin previously observed by the same authors. However, as the authors state, this result needs to be confirmed in patients who are obese asthmatics [42].

Finally, although there is growing evidence that designated obesity-related inflammation and secreted adipokines are possible mechanisms linking obesity and asthma, more studies are needed to allow us to elucidate the underlying mechanisms.

### 2.3. Other Proteins

Adipokines and cytokines secreted by the adipose tissue are not the only proteins that have emerged as key molecules in the development of asthma and obesity. Chitinase-3-like-1 (Chi3l1, YKL-40) a glycoprotein member of the glycosyl hydrolase 18 family, has been shown to play an important role in asthma-obesity development [43]. Using animal models of asthma and obesity, Ahangari and collaborators demonstrated that a high fat diet and Th2 inflammation induce Chi3l1 in visceral adipose tissue and pulmonary tissues, respectively. In the absence of Chi3l1, a blunted Th-2 response and decreased visceral fat accumulation have been observed. In asthmatic-obese subjects, Chi3l1 levels were positively associated with truncal adiposity and with persistent asthma and lung function [43]. In addition, higher Chi3l1 levels (168 ± 71.5 ng/mL) were associated with obesity-related asthma than with early-onset atopic and late-onset non-atopic phenotypes (80.62 ± 46.9 and 51.5 ± 24.9 ng/mL, respectively) [44].

### 2.4. Obesity, Asthma, and the Gut Microbiota

The large intestine contains an extremely dense population of microbes, collectively called gut microbiota. These microbes belong mainly to the Firmicutes, Bacteroidetes, Actinobacteria, Proteobacteria, Verrucomicrobia, and Fusobacteria phyla [45]. Altered ratios of Bacteroidetes and Firmicutes have been described in the obese state. However, there are contradicting data. Colon microbiota has a profound impact on human physiology and has a crucial role in human gastrointestinal health, modulating gastrointestinal tract development, metabolism and the immune system, and protecting against pathogens [46].

According to the “hygiene hypothesis” and the relevance of early life microbial exposures, the allergy epidemic has been attributed to factors that alter the gut microbiota [47]. The development of the microbiota takes place mainly during infancy, and in recent years there has been an exponential increase in the evidence of associations between the gut microbiota’s dysbiosis with diseases such as obesity and asthma [48]. Bacterial colonization is influenced by many factors, such as infant diet, mode of delivery (vaginal versus cesarean), and antibiotics. Indeed, we have described a differential clustering of bacterial components for infant gut microbiota based on the feeding method (breast-milk versus formula) [49]. It is important to note the recent discovery that within the first 6 weeks of life, the infant microbiota undergoes substantial reorganization, which is primarily driven by body site and not by the mode of delivery [50].

Different studies have pointed out a relationship among early antibiotic exposure and increased BMI in children. Antibiotics are a well-known factor that affects gut microbiota composition. A recent large international population-based cross-sectional study confirms the independent effect of early-life antibiotic use in promoting increased BMI among boys [51]. Treatment with perinatal vancomycin in a Th2 model of allergic asthma has important effects on disease severity, suggesting that alterations of the intestinal microbiota composition may be responsible for this effect. However, the same authors showed that severity of hypersensitivity pneumonitis was unaffected by vancomycin treatment [52]. Moreover, the use of antibiotics in children has been associated with marked changes in the intestinal microbiota composition that persist over 6 months. In particular, macrolide seems to modify the microbiota and its functions. Among children who received this antibiotic, a positive correlation between overall lifetime antibiotic use and BMI, as well as an increased risk of asthma, have been described, suggesting that macrolide may modify the microbiota in infants in a way that affects the weight gain associated with antibiotic use and asthma in later childhood [53].

One important function of the gut microbiota is maintaining intestinal integrity. In this regard, immunoglobulin A (IgA), the major class of antibody secreted by the gut mucosa, is an important factor. Secretoy IgA (sIgA) has a decisive function in the gut through its interaction with bacterial antigens, and also because it can limit the overgrowth of selected species, thus stimulating diversity. Children with allergic manifestations present a lower proportion of IgA at 12 months of age that may indicate impaired mucosal barrier function. [54]. In another study, high fecal calprotectin levels, an intestinal inflammation biomarker, at 2 months of age predicted the development of asthma and atopic dermatitis at the age of 6 years [55].

Among the functions of gut microbiota is fiber metabolization, raising the concentration of short chain fatty acids (SCFAs); mainly, acetate, butyrate, and propionate. It has been described that dietary fermentable fiber and SCFAs can outline the immunological lung environment and influence the severity of allergic inflammation [56]. Similarly, associations have been found between obesity and altered profiles of fecal SCFAs that could not be explained by microbial profiling alone. The production of SCFAs by the gut microbiota has been related to the development of asthma and obesity. SCFAs have been shown to be involved in different metabolic processes, such as the suppression of appetite, gluconeogenesis, fat metabolism, modulation of serotonin synthesis, and enhanced circadian rhythm, as well as having an anti-inflammatory role in the intestine. The widespread distribution of SCFAs receptors on a broad range of cells throughout the human body points to an untapped role for these molecules as therapeutic targets [47,57].

One important function of the gut microbiota is maintaining intestinal integrity. This contributes to the prevention of endotoxemia, a process resulting from translocation of LPS from gram-negative intestinal bacteria. LPS triggers a low-grade inflammatory response, and the process of endotoxemia can result in the development of insulin resistance and other metabolice diseases [57]; it also might impact asthma development. In an elegant study, Vatanen and collaborators discovered that Finnish and Estonian infants harbored both a greater proportion of Bacteroides species and enrichment in LPS biosynthesis-encoding genes when compared to Russian infants. Furthermore, their investigations pointed out that these Bacteroides species produced a structurally and functionally distinct form of LPS. This LPS differed from the LPS in the Russian microbiome, which was exclusively derived from *Escherichia coli*. Bacteroides LPS is structurally distinct from *Escherichia coli* LPS, and inhibits immune stimulation and the inflammatory cytokine response to *Escherichia coli* LPS in human cells. These findings suggest that differences in microbiota-derived LPS may preclude aspects of immune education [58].

Other important microbiota-derived metabolites are bile acids. The activation of bile acid-mediated signaling has been associated with an improvement in metabolic syndromes and enhanced control of inflammation. Bile acids inhibit Nod-like receptor family pyrin domain containing 3 (NLRP3) inflammasome activation through G protein-coupled bile acid receptor 1 (TGR5) and protein kinase A (PKA) activation. In vivo results showed that TGR5 receptor activation blocked NLRP3 inflammasome-dependent inflammation, including LPS systemic inflammation [59]. In asthmatic patients, higher plasma levels of taurine and bile acids, among other metabolites, have been described. It has been reported that nitric oxide (NO) modulates bile acid metabolism and bile production. It has been proposed that NO my increase bile acids because of increased hepatic synthesis, higher bacterial dehydroxylation in the gut, and increased conjugation in the liver [60,61].

In conclusion, the gut microbiota is associated with the development of immune disease. The underlying mechanisms are not yet fully understood, but LPS exposure, SCFAs production, and bile acids together with other gut microbiota-derived products might be implicated.

## 3. Materials and Methods

A comprehensive search of the relevant literature was performed across PubMed’s (U.S. National Library of Medicine and the National Institute of Health, Bethesda, MD, USA) MEDLINE (U.S. National Library of Medicine, Bethesda, MD, USA) over the last 5 years, using the following MeSH terms (Medical Subject Headings): “obesity” and “asthma” combined with “adipokines”, “phenotype”, “gastrointestinal microbiome”, and “inflammation”. Only articles based on scientific criteria and written in English were selected and assessed by manual and electronic means. Additionally, previous original articles and reviews focusing on epigenetics and microbiota in asthma and obesity were carefully examined, together with papers describing the role of SCFAs, LPS, and bile acids in obesity and asthma. A total of 183 articles were obtained, from which 46 were selected.

## 4. Conclusions

**Figure 2:**
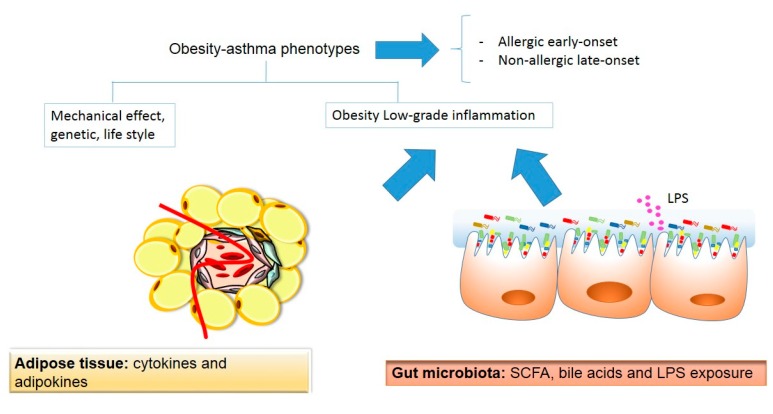
A schematic representation of the potential linking mechanisms between obesity and asthma. SCFAs: short-chain fatty acids; LPS: lipopolysaccharide. Beige color indicates adipose tissue origin, whereas the pink color indicates gut microbiota origin.

In the present review, we provided an overview of the potential mechanisms linking to the related conditions of obesity and asthma. A schematic representation of the linking mechanism between obesity and asthma is shown in Figure 2.

Two obesity-asthma phenotypes have been described: early-onset atopic asthma and late-onset nonatopic asthma. Mechanical, genetic, and lifestyle factors have been suggested as mediators in this association. Adipose tissue and gut microbiota can be considered an important player in both conditions. Adipose tissue secretes adipokines and cytokines that contribute to obesity-related low-grade inflammation, and might influence asthma development. Additionally, there has been recent growing interest in extracellular vesicles, particularly exosomes, as important mechanisms in metabolic homeostasis and crosstalk between different organs. Exosomes are membrane-bound nanovesicles, secreted from the endosomal pathway of cells, that contain protein and RNA, with an ability to target and modify specific cells and tissues. It has been proposed that exosomes released by adipocytes may contribute to tissue remodelling and systemic inflammation by reason of activated adipose tissue macrophages and endothelial cells and by modulating wide apart metabolic tissue [62]. However, this new and interesting field needs to be fully investigated. Recently, the gut microbiota has gained interest as a new factor in the development of both conditions. The gut microbiota can contribute to low-grade inflammation through the endotoxemia and the production of SCFAs and bile acids. High throughput sequencing technologies have allowed us to gain a better understanding of the composition and function of the gut microbiota. Nevertheless, more studies are needed to fully understand the association between both diseases. This includes basic, animal, and clinical studies, taking into account the different asthma phenotypes and using cutting edge techniques such as next-generation sequencing, metabolomics, and exosome studies.

## Abbreviations

BMI	Body Mass Index
AHR	Airway hyper-reactivity
LPS	Lipopolysaccharide
SCFAs	Short chain fatty acids
